# The Incidence and Propensity of Head Acceleration Events in a Season of Men’s and Women’s English Elite-Level Club Rugby Union Matches

**DOI:** 10.1007/s40279-024-02064-7

**Published:** 2024-06-26

**Authors:** David Allan, James Tooby, Lindsay Starling, Ross Tucker, Éanna Falvey, Danielle Salmon, James Brown, Sam Hudson, Keith Stokes, Ben Jones, Simon Kemp, Patrick O’Halloran, Matt Cross, Gregory Tierney

**Affiliations:** 1 Nanotechnology and Integrated Bioengineering Centre (NIBEC), School of Engineering, Ulster University, Belfast, UK; 2 Sport and Exercise Sciences Research Institute, Ulster University, Belfast, UK; 3 Carnegie Applied Rugby Research (CARR) Centre, Carnegie School of Sport, Leeds Beckett University, Leeds, UK; 4 World Rugby, 8-10 Pembroke St., Dublin, Ireland; 5 UK Collaborating Centre on Injury and Illness Prevention in Sport (UKCCIIS), University of Bath, Bath, UK; 6 Institute of Sport and Exercise Medicine, Stellenbosch University, Stellenbosch, South Africa; 7 School of Medicine and Health, University College Cork, Cork, Ireland; 8 Rugby Football Union, Twickenham, UK; 9 Premiership Rugby, London, UK; 10 England Performance Unit, Rugby Football League, Manchester, UK; 11 School of Behavioural and Health Sciences, Faculty of Health Sciences, Australian Catholic University, Brisbane, QLD Australia; 12 Division of Physiological Sciences and Health Through Physical Activity, Lifestyle and Sport Research Centre, Department of Human Biology, Faculty of Health Sciences, University of Cape Town, Cape Town, South Africa; 13 London School of Hygiene and Tropical Medicine, London, UK; 14 Sport and Exercise Medicine Service, University Hospitals Birmingham, Birmingham, UK; 15 Marker Diagnostics UK Ltd, Birmingham, UK

## Abstract

**Objectives:**

To describe and compare the incidence and propensity of head acceleration events (HAEs) using instrumented mouthguards (iMG) by playing position in a season of English elite-level men’s and women’s rugby union matches.

**Methods:**

iMG data were collected for 255 men and 133 women from 1,865 and 807 player-matches, respectively, and synchronised to video-coded match footage. Head peak resultant linear acceleration (PLA) and peak resultant angular acceleration (PAA) were extracted from each HAE. Mean incidence and propensity values were calculated across different recording thresholds for forwards and backs in addition to positional groups (front row, second row, back row, half backs, centres, back three) with 95% confidence intervals (CI) estimated. Significance was determined based on 95% CI not overlapping across recording thresholds.

**Results:**

For both men and women, HAE incidence was twice as high for forwards than backs across the majority of recording thresholds. HAE incidence and propensity were significantly lower in the women’s game compared to the men’s game. Back-row and front-row players had the highest incidence across all HAE thresholds for men’s forwards, while women’s forward positional groups and men’s and women’s back positional groups were similar. Tackles and carries exhibited a greater propensity to result in HAE for forward positional groups and the back three in the men’s game, and back row in the women’s game.

**Conclusion:**

These data offer valuable benchmark and comparative data for future research, HAE mitigation strategies, and management of HAE exposure in elite rugby players. Positional-specific differences in HAE incidence and propensity should be considered in future mitigation strategies.

**Supplementary Information:**

The online version contains supplementary material available at 10.1007/s40279-024-02064-7.

## Key Points

**Table:** 

Head acceleration event (HAE) mitigation strategies are a priority for rugby union to ensure brain health, in addition to player welfare, is optimised.
HAE incidence and propensity over a range of different thresholds have been reported in elite-level men’s and women’s rugby union for all positional groups (i.e. front row, second row, back row, half backs, centres, back three).
Positional-specific differences in HAE incidence and propensity exist and should be considered in any future HAE mitigation strategies.

## Introduction

The physical nature of contact sports such as rugby union means the risk of concussion and exposure to repetitive head acceleration events (HAEs) is inherent [1–3]. HAEs can result from either direct head contact or indirect (inertial) body contact [1, 4]. There is concern surrounding the potential medium and long-term health consequences of both concussions and repetitive HAEs [5–7]. Whilst the consequences of specific frequency and magnitudes of HAEs on long-term brain health remains unknown, a precautionary approach to reducing HAE exposure is recommended [1]. In the men’s game, the tackler accounts for most tackle-related concussions [8–10], whereas in the women’s game, the ball carrier appears as likely as the tackler to sustain a concussion [11, 12]. Differences in HAE mechanisms may, therefore, exist between the men’s and women’s games, and thus, sex-specific mitigation strategies may be needed.

Therefore, it is of paramount importance to develop strategies that aim to reduce both population and individual-level HAE exposure [9, 13, 14]. At the population level, HAE numbers may be reduced if exposure of all players to contact events is reduced by decreasing match numbers, limiting contact time in training, and reducing the frequency and number of contact events in matches through law changes [15–18]. On an individual level, HAE exposure may be decreased through individual player management (e.g. squad rotation that manages matches played per player per season) and interventions that improve technique in contact activities, such as tackles and rucks to reduce the likelihood of HAEs in at-risk players [9, 13, 14].

Head kinematics (linear acceleration, angular acceleration and angular velocity) are associated with both concussion injury risk and HAEs [1, 19, 20]. The primary contributor to brain deformation appears to be rotational head kinematics and various biomechanical brain injury mechanisms may exist, including those involving repetitive HAEs [1]. Some studies suggest that cumulative HAE exposure may lower an athlete’s tolerance to concussion [21, 22], and it has been postulated as a secondary injury mechanism [21]. Previous studies on HAEs in sports have been limited by low sample sizes and/or the validity of the biomechanical approaches undertaken (e.g., sensor-based approaches that suffer from soft tissue artefacts) [1, 23, 24]. Instrumented mouthguards (iMGs) can measure head linear and rotational kinematics on-field and are preferred for in vivo measurement of HAEs [23]. As a result, iMGs have been used in combination with qualitative video analysis in field-based studies involving rugby union, rugby league, and American football teams on an individual team basis or for a subset of matches [24–27].

Competition-wide implementation of iMGs in rugby union presents a unique opportunity to gain insights into HAE incidence and propensity and any differences that may exist between positions and sexes. The aim of this study was to describe the incidence and propensity of HAEs during elite-level men’s and women’s rugby union matches based on playing position.

## Methods

### Study Design and Participants

A prospective observational cohort study was undertaken with 255 men and 133 women. Participants were recruited from elite-level Premiership and Premier 15s clubs, respectively, during the 2022/23 season, which represent the highest levels of club rugby in England. Data were collected from domestic league, cup and European cup competitions in men (*n* = 1865 player-matches) and domestic league and cup competitions in women (*n* = 807 player-matches). All participants provided written consent, and ethical approval was obtained from the University’s research ethics committee, University of Ulster (#REC-21-0061). The participants underwent three-dimensional (3D) dental scans and received custom-fit iMG devices (Prevent Biometrics, Minneapolis, MN). These iMGs feature an accelerometer and gyroscope that sample at a rate of 3200 Hz, with measurement ranges of ± 200 g and ± 35 rad/s, respectively. Additionally, infra-red proximity sensors are embedded in the iMGs to assess their connection to the upper dentition during HAEs. Previous studies have validated the Prevent Biometrics iMGs, both in field and laboratory settings [28–31].

HAE events were identified when the linear acceleration exceeded 8 g on a single axis of the iMG accelerometer [27]. Kinematic data for HAEs were captured 10 ms before and 40 ms after the trigger event, with a recording threshold of 400 rad/s^2^ and 5 g at the head centre of gravity (CG) [27]. Peak resultant linear acceleration (PLA) at the head CG and peak resultant angular acceleration (PAA) of the head were extracted from each HAE. The level of noise/artefact in the kinematic signal was categorized into three classes (class 0 minimal signal noise, class 1 moderate signal noise and class 2 severe signal noise) using a Prevent Biometrics algorithm. A four-pole, zero-phase, low-pass Butterworth filter was applied to each signal, with cut-off frequencies of 200, 100 and 50 Hz for class 0, 1 and 2 HAEs, respectively, similar to previous studies [25, 27].

### Contact Event Identification

Tackle, carry and ruck contact events are video coded at the player-level by StatsPerform (Chicago, Illinois, United States) [27]. A custom MATLAB script was used to synchronise the timestamps of iMG HAEs with in-game video-coded contact events. The MATLAB script matched the HAE impact time (universal time coordinated; UTC) with in-game video timestamps from commercially available match data provided by StatsPerform, along with broadcast-quality game footage. A subset of HAEs (*n* = 1210) was manually video analysed to test the accuracy of the MATLAB script. The MATLAB script correctly linked 88% of HAEs to the StatsPerform contact events when compared with the manual video analysis approach. As a 400 rad/s^2^ and 5 g threshold was used, the number of false positive events, i.e. events that did not originate from contact, is expected to be very low (positive predictive value > 0.99) [27]. Only contact events that had HAEs linked to video-coded events were used for the purposes of calculating HAE propensity. All HAEs captured during the match period, including HAEs that were unpaired to a tackle, carry, ruck, or any other contact event, were used for incidence calculations.

### Statistical Analysis

Incidence was calculated on a per-player basis as the number of HAEs per player-hour [27]. Playing time for each player was obtained from data provided by StatsPerform for each player-match. Propensity values were calculated on a per-player basis by dividing the number of each contact event type that resulted in an HAE by the total number of each contact event type the player was involved in while wearing an iMG [27]. Only contact events that corresponded with an on-the-teeth period (based on the iMG proximity sensor) for the instrumented player were used in propensity calculations, and only player-matches where the instrumented player wore their iMG for a minimum of 90% of their contact events were used in the incidence calculations [27]. Mean incidence and propensity values were calculated across different recording thresholds for forwards and backs in addition to positional groups (front row, second row, back row, half backs, centres, back three) with 95% confidence interval (CI) estimated using a bootstrapping procedure [27]. Significance was determined on the basis of 95% CI not overlapping across recording thresholds [27].

## Results

### General

HAEs were captured from 178 individual players across 1127 individual player-games for the men’s game. Of these, 4931 tackles, 3189 carries and 4084 rucks had at least one HAE associated with the contact event. Overall median PLA values for tackles, carries and rucks were 14.9 g (Q1 = 10.1 g, Q3 = 22.7 g), 15.7 g (Q1 = 11.0 g, Q3 = 24.0 g) and 14.7 g (Q1 = 10.3 g, Q3 = 21.7 g), respectively (Table 1).

**Table 1:** Breakdown of the number of contact events (*n*) for tackles, carries as rucks broken down by main positional groups (forwards and backs) in addition to sub-positional groups for the men’s game

	*n*	Median	Q1–Q3	95th		*n*	Median	Q1–Q3	95th		*n*	Median	Q1–Q3	95th
Tackles	4931	14.9 g	10.1–22.7 g	43.8 g	Forwards	3595	14.7 g	10.1–22.3 g	41.6 g	Front row	1141	15.6 g	10.9–23.1 g	42.0 g
										Second row	1124	12.7 g	9.2–20.1 g	35.9 g
										Back row	1330	16.0 g	10.3–24.2 g	45.1 g
					Backs	1336	15.4 g	10.5–24.1 g	46.5 g	Half back	257	14.3 g	10.0–22.4 g	46.6 g
										Centre	531	15.8 g	10.9–24.5 g	48.8 g
										Back three	548	15.7 g	10.3–24.5 g	44.4 g
Carries	3189	15.7 g	11.0–24.0 g	41.0 g	Forwards	2010	16.1 g	11.1–24.5 g	41.1 g	Front row	581	16.5 g	11.5–25.5 g	41.3 g
										Second row	670	15.0 g	11.0–23.6 g	40.5 g
										Back row	759	16.4 g	11.1–24.9 g	41.1 g
					Backs	1179	15.2 g	10.7–23.0 g	40.3 g	Half back	106	15.8 g	10.7–23.5 g	41.1 g
										Centre	359	15.1 g	10.6–23.9 g	43.6 g
										Back three	714	15.1 g	10.8–22.6 g	39.7 g
Rucks	4084	14.7 g	10.3–21.7 g	39.1 g	Forwards	3118	14.5 g	10.3–21.7 g	39.3 g	Front row	989	14.8 g	10.4–21.3 g	37.3 g
										Second row	871	13.9 g	10.2–20.2 g	35.3 g
										Back row	1258	14.9 g	10.5–23.0 g	42.2 g
					Backs	966	15.2 g	10.2–21.9 g	38.0 g	Half back	100	15.3 g	9.6–21.1 g	29.2 g
										Centre	394	15.3 g	10.8–22.6 g	38.4 g
										Back three	472	14.9 g	9.9–21.3 g	39.7 g

For each sub-position, main group and overall, median, interquartile range and 95th percentile for PLA are also presented. Supplementary Table 1 presents the results for PAA

HAEs were captured from 107 individual players across 464 individual player-games for the women’s game. Of these, 1383 tackles, 732 carries and 775 rucks had at least one HAE associated with the contact event. Overall median PLA values for tackles, carries and rucks were 12.4 g (Q1 = 8.8 g, Q3 = 18.6 g), 12.2 g (Q1 = 9.1 g, Q3 = 17.9 g) and 12.7 g (Q1 = 9.0 g, Q3 = 19.2 g) respectively (Table 2).

**Table 2:** Breakdown of the number of contact events (*n*) for tackles, carries as rucks broken down by main positional groups (forwards and backs) and sub-positional groups for the women’s game

	*n*	Median	Q1–Q3	95th		*n*	Median	Q1–Q3	95th		*n*	Median	Q1–Q3	95th
Tackles	1383	12.4 g	8.8–18.6 g	35.3 g	Forwards	864	12.6 g	8.9–18.5 g	33.9 g	Front row	170	12.0 g	8.0–17.1 g	36.3 g
										Second row	395	12.2 g	8.8–18.0 g	31.8 g
										Back row	299	13.7 g	9.3–19.4 g	34.2 g
					Backs	519	12.0 g	8.6–19.3 g	38.6 g	Half back	100	10.9 g	7.7–19.1 g	36.2 g
										Centre	293	12.8 g	9.1–19.3 g	35.6 g
										Back three	126	11.7 g	8.5–17.1 g	43.3 g
Carries	732	12.2 g	9.1–17.9 g	31.3 g	Forwards	477	12.5 g	9.4–18.5 g	32.8 g	Front row	91	12.2 g	9.5–18.9 g	32.9 g
										Second row	187	13.7 g	9.2–18.9 g	31.4 g
										Back row	199	12.4 g	9.6–17.8 g	33.1 g
					Backs	255	11.6 g	8.6–16.7 g	27.4 g	Half back	30	11.8 g	9.4–17.3 g	50.5 g
										Centre	128	11.9 g	8.5–16.8 g	24.5 g
										Back three	97	11.3 g	8.7–15.4 g	28.1 g
Rucks	775	12.7 g	9.0–19.2 g	31.8 g	Forwards	580	13.1 g	9.2–19.1 g	32.2 g	Front row	152	12.9 g	9.6–17.9 g	31.4 g
										Second row	240	12.4 g	8.6–18.9 g	33.1 g
										Back row	188	14.1 g	9.9–20.7 g	31.6 g
					Backs	195	12.0 g	8.3–19.7 g	31.2 g	Half back	34	11.6 g	8.8–15.0 g	26.6 g
										Centre	108	14.4 g	8.8–21.9 g	33.7 g
										Back three	53	10.1 g	7.6–16.6 g	26.7 g

For each sub-position, main group and overall, median, interquartile range and 95th percentile for PLA are also presented. Supplementary Table 2 presents the results for PAA

### Men’s HAE Incidence

**Fig. 1:**
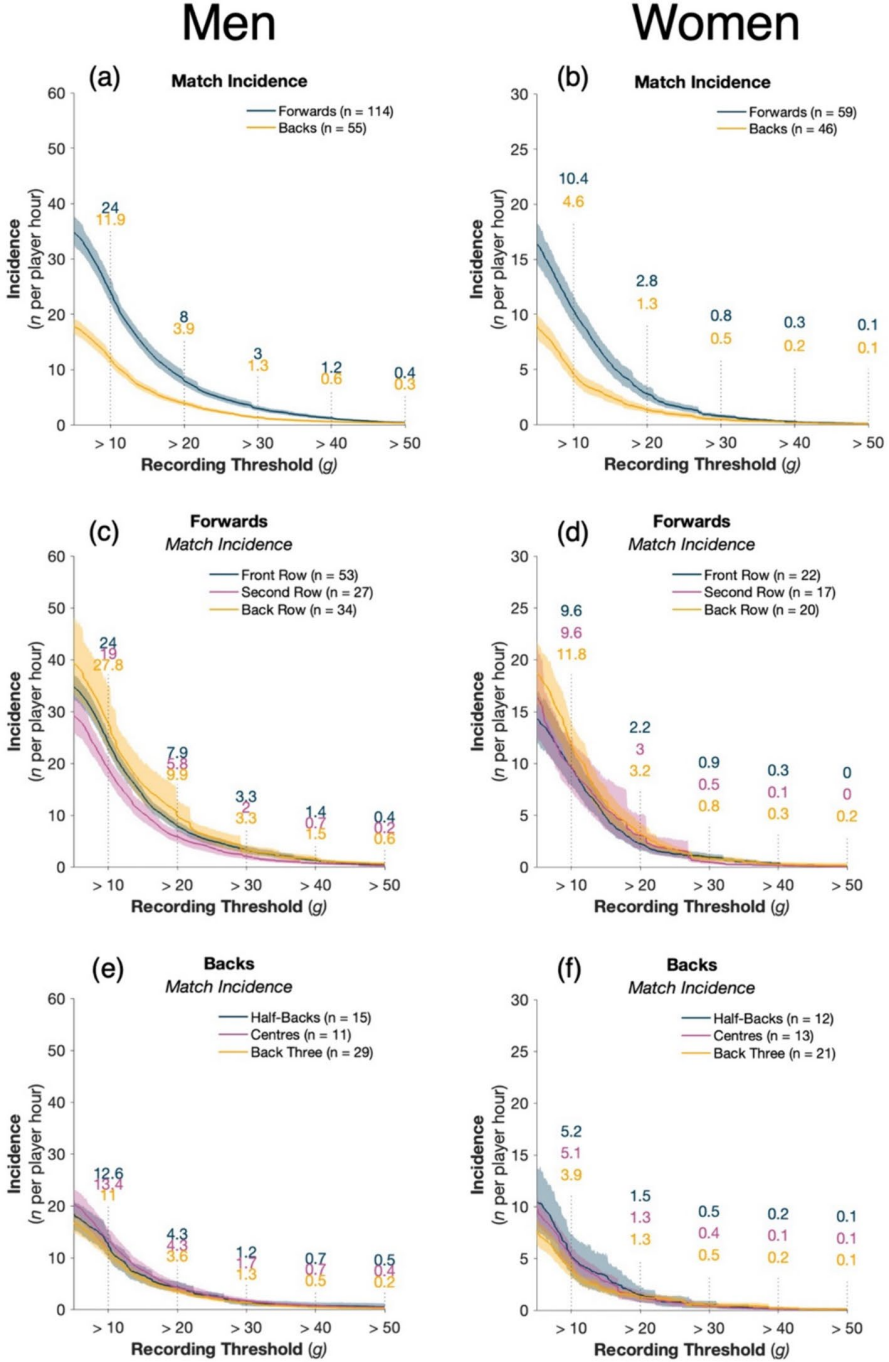
HAE incidence for men and women as PLA recording threshold increases (**a**, **b**). HAE incidence for the forward (**c**, **d**) and back (**e**, **f**) positional groups. Shaded regions indicate 95% CI and *n* represents the number of players available for calculation based on compliance requirements (Sect. 2). Supplementary Fig. 1 presents the results with a PAA recording threshold

Forwards experienced, on average, twice as many HAEs per player-hour across the range of HAE thresholds compared with backs (Fig. 1a). Within the forward group (front row, second row, back row), the back row had the highest incidence per player-hour across all HAE thresholds, with the second row experiencing the lowest incidence across all HAE thresholds (Fig. 1c). In comparison, positions within the backs had similar incidence at all thresholds (Fig. 1e).

### Women’s HAE Incidence

HAE incidence was twice as high in forwards compared with backs across the range of HAE thresholds examined (Fig. 1b). All positional groups within the forwards (front row, second row, back row), had a similar HAE incidence across all HAE thresholds (Fig. 1d). Similarly, among the backs (half-backs, centres, back three), incidence was similar across all thresholds (Fig. 1f).

### Men’s HAE Propensity

**Fig. 2:**
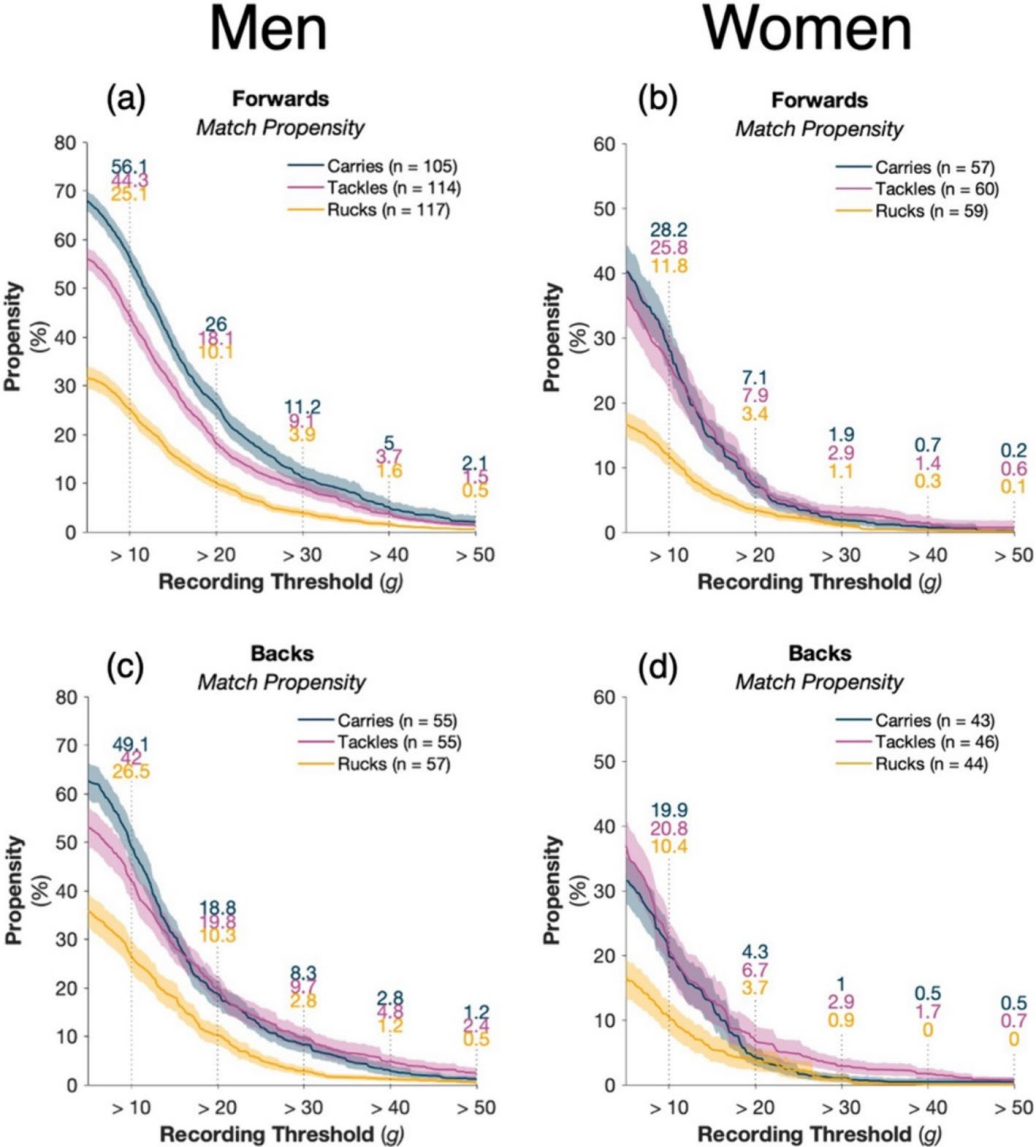
The propensity of tackles, carries and rucks for men and women to result in at least one HAE exceeding a given magnitude as PLA recording threshold increases for forwards (**a**, **b**) and backs (**c**, **d**). Shaded regions indicate 95% CI, and *n* represents the number of players available for calculation based on compliance requirements (Sect. 2). Supplementary Fig. 2 presents the results with a PAA recording threshold

In forwards, 55.7% of tackles, 43.9% of carries and 74.9% of ruck events did not exceed 10 g (Fig. 2a). In backs, 58.0% of tackles, 50.9% of carries, and 73.5% of rucks did not exceed 10 g (Fig. 2c). The propensity for tackles to result in HAEs between 10 and 20 g was 26.2% in forwards and 22.2% in backs, compared with 2.2% in forwards and 2.4% in backs for HAEs between 40 and 50 g (Fig. 2a, c). Additionally, forwards had a greater HAE propensity from carries and/or tackles than rucks (Fig. 2a). For backs, HAE propensity was greater for tackles and carries than rucks across all thresholds (Fig. 2c).

**Fig. 3:**
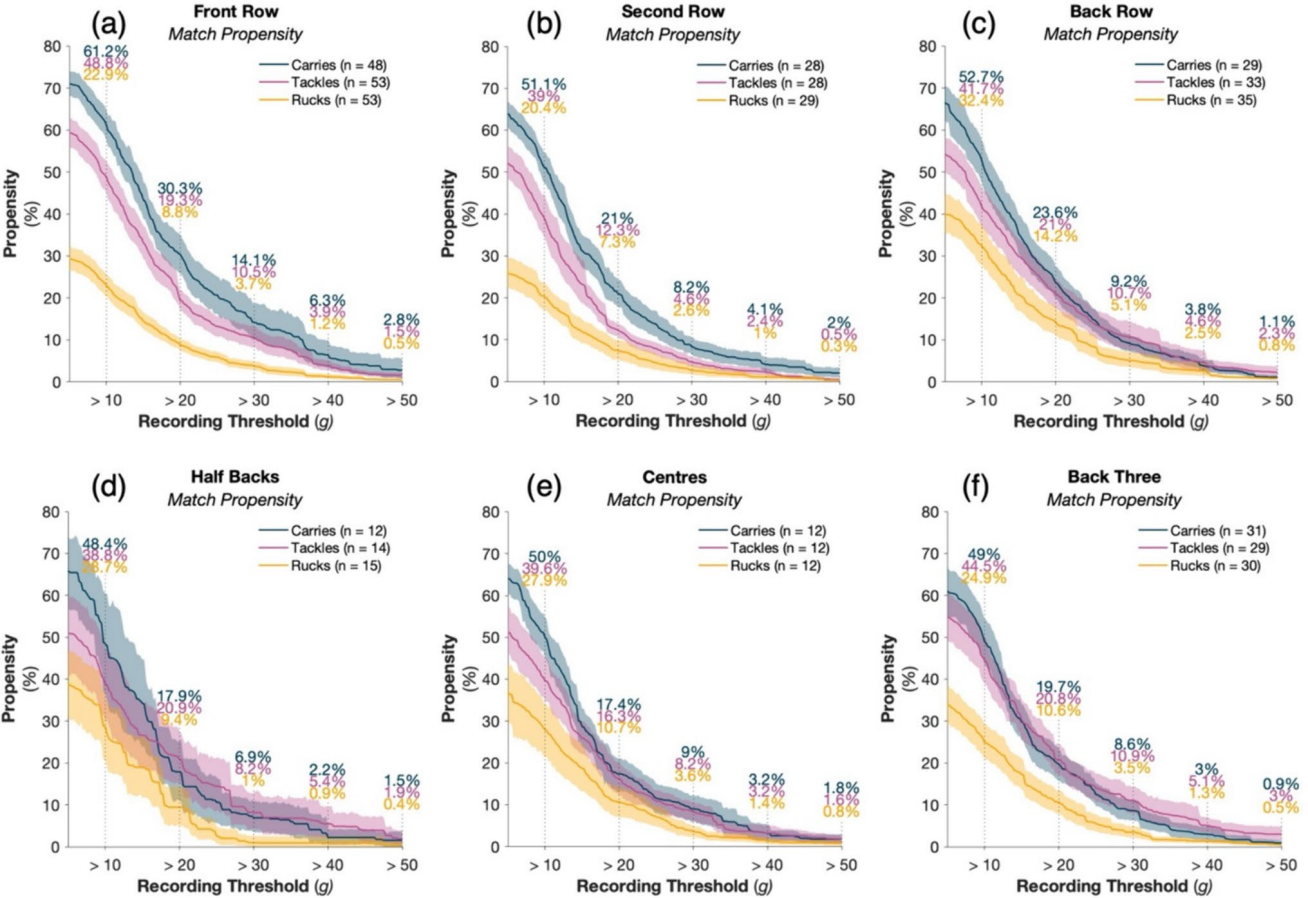
The propensity of tackles, carries and rucks for the men’s game to result in at least one HAE exceeding a given magnitude as PLA recording threshold increases for the front row (**a**), second row (**b**), back row (**c**), half backs (**d**), centres (**e**) and back three (**f**) positional groups. Shaded regions indicate 95% CI and *n* represents the number of players available for calculation based on compliance requirements (Sect. 2). Supplementary Fig. 3 presents the results with a PAA recording threshold

The propensity for tackles to result in HAEs between 10 and 20g was 29.5% for the front-row, 26.7% for the second-row, and 20.7% for the back-row (Fig. 3). The propensity for tackles to result in HAEs between 40 and 50 g was 2.4, 1.9 and 2.3% in front-row, second row and back-row players, respectively. The propensity for tackles to result in HAEs between 10 and 20 g was 17.9% for the half-backs, 23.3% for the centres and 23.7% for the back three. Between 40 and 50 g, tackle propensity was 3.5, 1.6 and 2.1% for the same respective positional groups. Across all positional groups, the HAE propensity at rucks was lower than that of tackles and/or carries (Fig. 3).

### Women’s HAE Propensity

In forwards, 74.2% of tackles, 71.8% of carries and 88.2% of ruck events did not exceed 10 g (Fig. 2b), with 79.3% of tackles, 80.1% of carries and 89.6% of rucks not exceeding 10 g for backs (Fig. 2d). The propensity of tackles to result in HAEs between 10 and 20 g was 17.9% for forwards and 14.1% for backs, compared with 0.8% and 1% of tackles to result in HAE between 40 and 50 g (Fig. 2b, d). Forwards and backs had a greater HAE propensity from tackles and/or carries compared with rucks (Fig. 2).

**Fig. 4:**
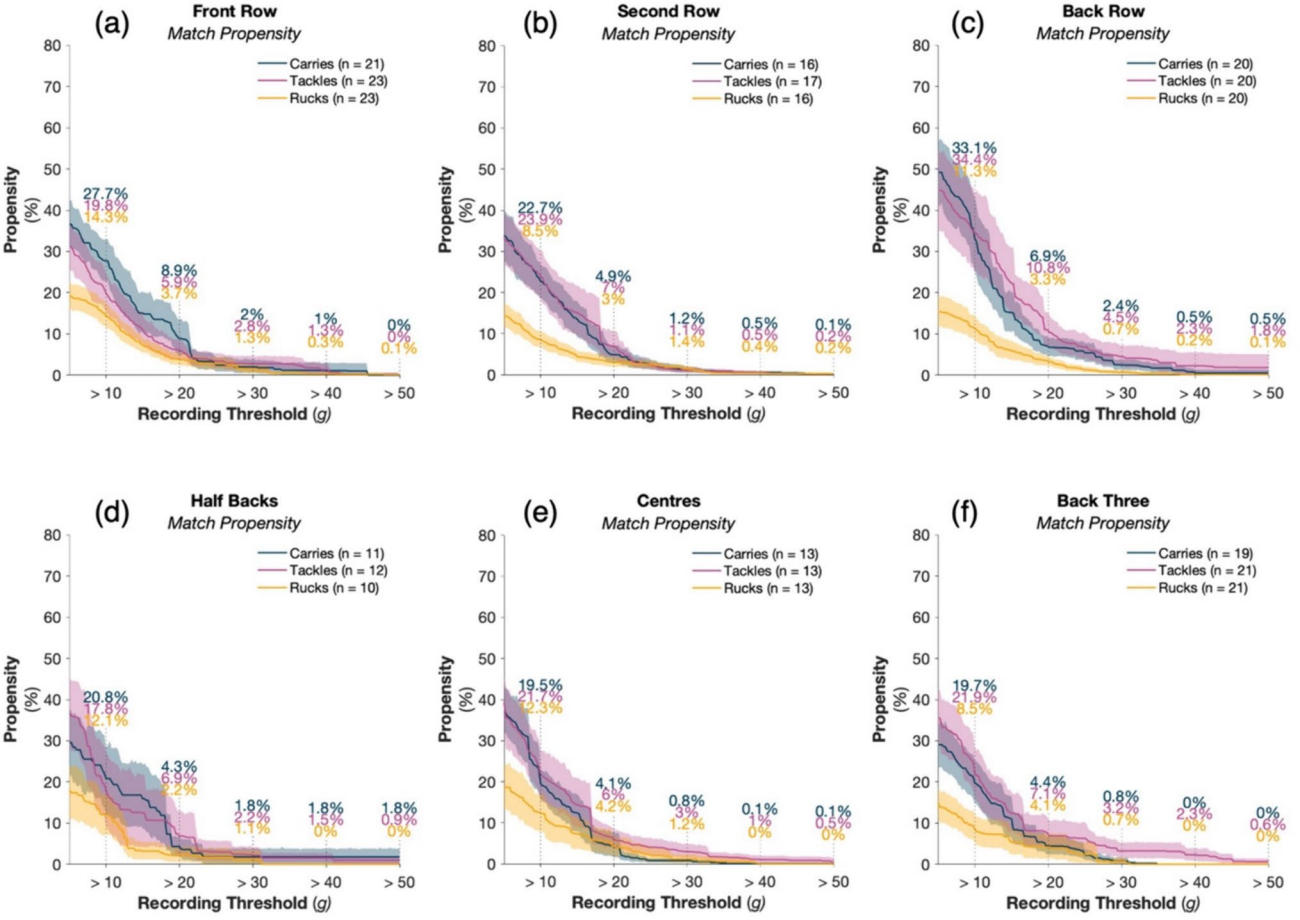
The propensity of tackles, carries and rucks for the women’s game to result in at least one HAE exceeding a given magnitude as PLA recording threshold increases for the front row (**a**), second row (**b**), back row (**c**), half backs (**d**), centres (**e**) and back three (**f**) positional groups. Shaded regions indicate 95% CI and *n* represents the number of players available for calculation based on compliance requirements (Sect. 2). Supplementary Fig. 4 presents the results with a PAA recording threshold

For positional groups, 13.9% of tackles produced an HAE between 10 and 20 g in the front-row, compared with 16.9% for the second-row, and 23.6% for the back-row (Fig. 4). At higher HAE magnitudes, between 40 and 50 g, propensity was 1.3, 0.3 and 0.5% in front-row, second-row and back-row players, respectively.

## Discussion

### General

This study describes HAE incidence and propensity data for men and women that will be used for benchmarking purposes as iMG use becomes more widespread in elite rugby union. This data will assist with the identification of mechanistic factors and inform the development and evaluation of mitigation strategies aimed at reducing HAE exposure in the sport and their effectiveness. The HAE incidence and propensity across recording thresholds is very similar to pilot findings in elite rugby union that also found HAE incidence was greater in forwards compared with backs for both sexes and in men compared with women [27]. Our contribution was to explore how competition-wide, season-long HAE incidence and propensity were affected by playing positional groups, and to describe the high-level match activity that was responsible for HAE across positional groups.

Our findings have a number of important implications for stakeholders in the sport in relation to understanding and managing both the short- and potential longer-term effects on brain health that may exist as a result of cumulative exposure to HAEs [7]. This includes the ability to track and compare HAE incidence and number in players over time. This can, in turn, inform and guide the management of individual players’ HAE exposure in matches. It may also help to identify mitigation strategies that can be specifically targeted at contact behaviours that increase HAE risk for players and/or positional groups in whom HAEs occur more frequently or at a greater rate. These mitigation strategies could also be tailored to the level of play where contact characteristics may vary.

### HAE Incidence by Positional Group

Forwards have an HAE incidence that is twice as high as that in backs in both men and women. A previous study found that this is the result of exposure to a greater number of contact events and not the propensity of contact events to cause HAEs [27]. Given the focus on reducing HAE exposure for the risk reduction this confers, the management of match HAEs in forwards is of paramount importance, and this may be achieved by means of managing exposure (or “workload”) in matches. More specifically, within the men’s forward group, front/back row players had the highest incidence per player-hour across all HAE thresholds, whereas all positional groups within the backs had a similar incidence at all thresholds with an overall lower incidence rate. Any HAE exposure management strategies should account for positional group differences.

### Match Activities and HAE Propensity

HAE propensity was significantly lower in the women’s game compared to the men’s game. The distribution of acceleration events favours lower acceleration events in the women’s game compared with the men’s game when looking at the propensity of HAE events over 10 g (Fig. 2). The mechanisms that result in this difference in propensity between men and women require future mechanistic studies, with speculation that these differences may be a result of physical/technique differences between men’s and women’s contact events [27, 32]. This may have implications for how HAEs are measured moving forward, particularly since the clinical implications of HAEs at different magnitudes are unknown. Therefore, it may be prudent to quantify HAEs against different thresholds in men compared to women, such that HAEs are matched for number (the outcome) rather than the threshold HAE magnitude.

Previous research on the mechanism of head injuries and concussions has found that in men’s rugby union, suspected/confirmed concussion risk was significantly greater for the tackler than the ball carrier [2, 10]. We have measured HAE propensity and incidence rather than clinical outcomes and found that HAE propensity for carries was similar to or greater than that of tackles in forwards (Fig. 2).

Tackle events, compared with rucks and carries, have a greater propensity to result in higher magnitude HAEs for women backs, which may suggest that technique and/or situational differences play a role and position-specific mitigation and/or coaching strategies are needed [11, 32–34]. Nevertheless, the tackle event, which involves tackler(s) and ball carrier, remains the predominant source of HAEs in the game. Any strategies that reduce HAE numbers should, in theory, address the tackle as the main target of risk reduction and subsequently reduce the number of head injury assessments (HIAs) and concussions [8–10, 13, 35]. However, the specific risk reductions to the tackler and ball carrier may vary [8]. Therefore, monitoring and linking both HIA1, concussion and HAE incidence after implementing mitigation policies is crucial for gaining a deeper understanding of how HAEs contribute to clinical outcomes and identifying any potential unintended consequences that may arise when attempting to mitigate one outcome while affecting the other.

### Limitations

One of the main limitations of this study is that it does not consider the cumulative effect of additional impacts and subsequent HAEs that occur throughout training sessions. The inclusion of training data was initial considered by the author group however, after assessing the quality of the training data acquired, individual player training data was deemed to be too inconsistent and of insufficient quality to provide any additional meaningful insight into the effect of these training HAEs. It is acknowledged that when developing future HAE reduction strategies, the effect of HAEs sustained during training must be considered.

Player compliance with iMG wearing additionally posed a significant challenge over the duration of this study. Out of the 530 and 232 men and women participants who consented to participate and were provided with an iMG, data was collected from 255 and 133 individual players, respectively. This may bias the study results since players wearing the iMG, whose data are presented here, may not necessarily represent all players in the sport. The iMG utilised within this study demonstrated high scores for mouthguard fit, mouthguard comfort and practitioner usability [31]. Future research should investigate issues surrounding player compliance.

This study did not investigate the influence of specific tackle, carry or ruck techniques, or other more detailed characteristics of these contact events, on HAE incidence and propensity. Technique is recognised to be a significant risk factor for injuries, including concussion [14, 36]. Future research should assess the influence of technique on HAE incidence and propensity, as well as seeking to identify the characteristics of tackles and rucks that increase HAE risk. This understanding will benefit the refinement of technical coaching strategies and/or influence possible law changes in the sport to reduce HAE numbers, and by extension, injury risk. Other contact events such as scrums and their propensity to result in HAE should be investigated.

While this study represents the largest dataset on elite men’s and women’s HAEs to date, it may not comprehensively reflect the diverse playing styles and conditions encountered within rugby union globally. Consequently, the incidence and predisposition to HAEs may vary in other rugby populations, particularly in youth and community games. The contribution of training on HAE exposure warrants further exploration.

Finally, it is important to note that this study focussed on peak resultant head kinematics (PLA, PAA) but did not consider factors such as directionality and temporal aspects, such as pulse duration, of the kinematic signals recorded by the iMG. Temporal and directional elements are likely crucial for understanding injury risk and should be considered for inclusion in future research, particularly with respects to the relationship between HAE magnitude and clinical outcomes. The filtering of kinematics was conducted by Prevent Biometrics in-house processes which has been incorporated into previous validations of the iMG system [31]. However, a common signal and data processing approach for iMG systems is warranted.

## Conclusion

Season-long implementation of iMG was undertaken across a men’s and women’s elite-level club competition. Forwards had a greater HAE incidence than backs in both the men’s and women’s game. Back row and front row players had the highest incidence for men’s forwards positional groups. HAE incidence in women’s forward positional groups and men’s and women’s back positional groups appeared similar. Tackles and carries exhibited a greater propensity to result in HAE for forward positional groups and the back three in men’s and back rows in women’s game. These findings offer valuable positional group-specific benchmark and comparative data for future research, for HAE mitigation strategies and for management of HAE exposure in elite rugby players. Positional-specific differences in HAE incidence and propensity should be considered in any future mitigation strategies.

## Policy Implications

The results of this study provide competition-wide and season-long match HAE incidence and propensity data that offers valuable benchmark data for stakeholders regarding match HAE exposure and comparative data for future research assessing the efficacy of mitigation strategies. This study also suggests that positional group-specific risk mitigation strategies, specifically for forwards, are likely to be necessary.

## Supplementary Information

Below is the link to the electronic supplementary material.

Supplementary file1 (DOCX 1158 KB)

## Declarations

### Data sharing

Anonymised data available upon reasonable request.

### Patient and public involvement

Patients and/or the public were not involved in the design, conduct, reporting, or dissemination plans of this research.
