# Supplementary material for: The Incidence and Propensity of Head Acceleration Events in a Season of Men’s and Women’s English Elite-Level Club Rugby Union Matches
Source: Sports Med. 2024 Jun 26;54(10):2685–96. doi: 10.1007/s40279-024-02064-7 (PMC11467118; doi:10.1007/s40279-024-02064-7)
Supplement: Supplementary file 1 — Supplementary file1 (DOCX 1158 KB) [file 40279_2024_2064_MOESM1_ESM.docx]

**The incidence and propensity of head acceleration events in a season of men’s and women’s English elite-level club rugby union matches.**

**Sports Medicine**

David Allan^1,2^, James Tooby^3^, Lindsay Starling ^4,5^, Ross Tucker^4,6^, Éanna Falvey^4,7^, Danielle Salmon^4^, James Brown^3,6^, Sam Hudson^5^, Keith Stokes^5,8^, Ben Jones,^3,9,10,11,12^, Simon Kemp^8,13^, Patrick O’Halloran^14,15^, Matt Cross^3,9^, Gregory Tierney^1,2^

^1^ Nanotechnology and Integrated Bioengineering Centre (NIBEC), School of Engineering, Ulster University, Belfast, United Kingdom

^2^ Sport and Exercise Sciences Research Institute, Ulster University, Belfast, United Kingdom

^3^ Carnegie Applied Rugby Research (CARR) Centre, Carnegie School of Sport, Leeds Beckett University, Leeds, United Kingdom

^4^ World Rugby, 8-10 Pembroke St., Dublin, Ireland

^5^ UK Collaborating Centre on Injury and Illness Prevention in Sport (UKCCIIS), University of Bath, United Kingdom

^6^ Institute of Sport and Exercise Medicine, Stellenbosch University, South Africa

^7^ School of Medicine & Health, University College Cork, Cork, Ireland

^8^ Rugby Football Union, Twickenham, United Kingdom

^9^ Premiership Rugby, London, United Kingdom

^10^ England Performance Unit, Rugby Football League, Manchester, United Kingdom

^11^ School of Behavioural and Health Sciences, Faculty of Health Sciences, Australian Catholic University, Brisbane, QLD, Australia

^12^ Division of Physiological Sciences and Health through Physical Activity, Lifestyle and Sport Research Centre, Department of Human Biology, Faculty of Health Sciences, University of Cape Town, Cape Town, South Africa

^13^ London School of Hygiene and Tropical Medicine, London, United Kingdom

^14^ Sport and Exercise Medicine Service, University Hospitals Birmingham, United Kingdom

^15^ Marker Diagnostics UK Ltd, United Kingdom

**Corresponding author:** Dr David Allan, Ulster University, Belfast, United Kingdom. Email: D.Allan@Ulster.ac.uk

**Supplementary Tables**

Supplementary Table 1: Breakdown of the number of contact events (n) for tackles, carries as rucks broken down by main positional groups (forwards and backs) in addition to sub-positional groups for the men’s game. For each sub-position, main group, and overall, median, interquartile range, and 95^th^ percentile for PAA are also presented.

|  | n | Median (rad/s^2^) | Q1-Q3  (rad/s^2^) | 95^th^  (rad/s^2^) |  | n | Median  (rad/s^2^) | Q1-Q3  (rad/s^2^) | 95^th^  (rad/s^2^) |  | n | Median  (rad/s^2^) | Q1-Q3  (rad/s^2^) | 95^th^  (rad/s^2^) |
| --- | --- | --- | --- | --- | --- | --- | --- | --- | --- | --- | --- | --- | --- | --- |
| **Tackles** | 4931 | 1099 | 742-1679 | 3088 | **Forwards** | 3595 | 1077 | 728-1628 | 3027 | **Front Row** | 1141 | 1092 | 748-1643 | 3103 |
|  |  |  |  |  |  |  |  |  |  | **Second Row** | 1124 | 987 | 682-1504 | 2650 |
|  |  |  |  |  |  |  |  |  |  | **Back Row** | 1330 | 1150 | 769-1745 | 3119 |
|  |  |  |  |  | **Backs** | 1336 | 1189 | 780-1787 | 3290 | **Half Back** | 257 | 1154 | 704-1767 | 3446 |
|  |  |  |  |  |  |  |  |  |  | **Centre** | 531 | 1250 | 835-1888 | 3310 |
|  |  |  |  |  |  |  |  |  |  | **Back Three** | 548 | 1125 | 781-1722 | 3213 |
| **Carries** | 3189 | 1085 | 738-1630 | 2872 | **Forwards** | 2010 | 1092 | 738-1624 | 2800 | **Front Row** | 581 | 1151 | 760-1601 | 2927 |
|  |  |  |  |  |  |  |  |  |  | **Second Row** | 670 | 1055 | 729-1561 | 2591 |
|  |  |  |  |  |  |  |  |  |  | **Back Row** | 759 | 1094 | 736-1693 | 2870 |
|  |  |  |  |  | **Backs** | 1179 | 1077 | 738-1635 | 2984 | **Half Back** | 106 | 1124 | 772-1688 | 2955 |
|  |  |  |  |  |  |  |  |  |  | **Centre** | 359 | 1080 | 752-1630 | 3202 |
|  |  |  |  |  |  |  |  |  |  | **Back Three** | 714 | 1054 | 733-1634 | 2828 |
| **Rucks** | 4084 | 985 | 677-1476 | 2711 | **Forwards** | 3118 | 971 | 666-1463 | 2748 | **Front Row** | 989 | 945 | 648-1455 | 2591 |
|  |  |  |  |  |  |  |  |  |  | **Second Row** | 871 | 936 | 656-1378 | 2653 |
|  |  |  |  |  |  |  |  |  |  | **Back Row** | 1258 | 1021 | 689-1511 | 2912 |
|  |  |  |  |  | **Backs** | 966 | 1032 | 699-1547 | 2681 | **Half Back** | 100 | 1002 | 676-1406 | 2842 |
|  |  |  |  |  |  |  |  |  |  | **Centre** | 394 | 1061 | 713-1631 | 2625 |
|  |  |  |  |  |  |  |  |  |  | **Back Three** | 472 | 990 | 694-1456 | 2703 |

Supplementary Table 2: Breakdown of the number of contact events (n) for tackles, carries as rucks broken down by main positional groups (forwards and backs) in addition to sub-positional groups for the women’s game. For each sub-position, main group, and overall, median, interquartile range, and 95^th^ for PAA are also presented.

|  | n | Median (rad/s^2^) | Q1-Q3  (rad/s^2^) | 95^th^  (rad/s^2^) |  | n | Median  (rad/s^2^) | Q1-Q3  (rad/s^2^) | 95^th^  (rad/s^2^) |  | n | Median  (rad/s^2^) | Q1-Q3  (rad/s^2^) | 95^th^  (rad/s^2^) |
| --- | --- | --- | --- | --- | --- | --- | --- | --- | --- | --- | --- | --- | --- | --- |
| **Tackles** | 1383 | 1017 | 683-1530 | 2625 | **Forwards** | 864 | 1024 | 679-1505 | 2534 | **Front Row** | 170 | 946 | 677-1396 | 2428 |
|  |  |  |  |  |  |  |  |  |  | **Second Row** | 395 | 1039 | 689-1489 | 2563 |
|  |  |  |  |  |  |  |  |  |  | **Back Row** | 299 | 1037 | 670-1573 | 2563 |
|  |  |  |  |  | **Backs** | 519 | 1004 | 687-1556 | 2722 | **Half Back** | 100 | 1006 | 668-1556 | 2835 |
|  |  |  |  |  |  |  |  |  |  | **Centre** | 293 | 1008 | 716-1503 | 2607 |
|  |  |  |  |  |  |  |  |  |  | **Back Three** | 126 | 995 | 642-1613 | 2738 |
| **Carries** | 732 | 927 | 664-1407 | 2655 | **Forwards** | 477 | 952 | 683-1445 | 2738 | **Front Row** | 91 | 921 | 705-1403 | 2422 |
|  |  |  |  |  |  |  |  |  |  | **Second Row** | 187 | 1035 | 681-1554 | 2440 |
|  |  |  |  |  |  |  |  |  |  | **Back Row** | 199 | 865 | 671-1379 | 2793 |
|  |  |  |  |  | **Backs** | 255 | 880 | 633-1305 | 2248 | **Half Back** | 30 | 944 | 762-1419 | 2883 |
|  |  |  |  |  |  |  |  |  |  | **Centre** | 128 | 865 | 617-1298 | 2329 |
|  |  |  |  |  |  |  |  |  |  | **Back Three** | 97 | 835 | 628-1294 | 2071 |
| **Rucks** | 775 | 955 | 657-1402 | 2540 | **Forwards** | 580 | 954 | 656-1392 | 2486 | **Front Row** | 152 | 935 | 682-1334 | 2717 |
|  |  |  |  |  |  |  |  |  |  | **Second Row** | 240 | 901 | 621-1363 | 2405 |
|  |  |  |  |  |  |  |  |  |  | **Back Row** | 188 | 1002 | 702-1489 | 2303 |
|  |  |  |  |  | **Backs** | 195 | 982 | 659-1483 | 2598 | **Half Back** | 34 | 1086 | 688-1565 | 2380 |
|  |  |  |  |  |  |  |  |  |  | **Centre** | 108 | 1089 | 663-1617 | 2679 |
|  |  |  |  |  |  |  |  |  |  | **Back Three** | 53 | 828 | 597-1300 | 2945 |

**Supplementary Figures**

**
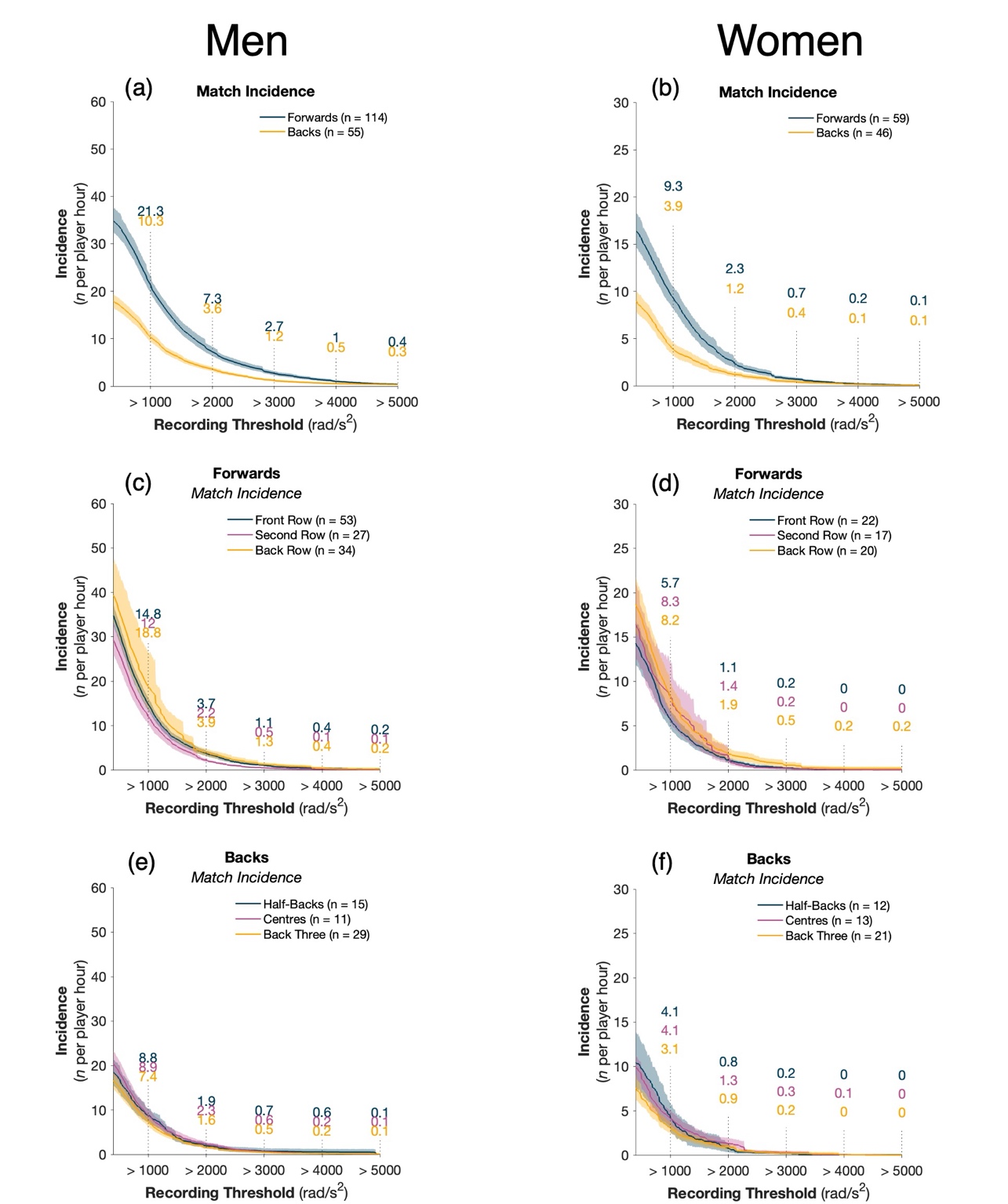
**

Supplementary Figure 1. HAE incidence for men and women as PAA recording threshold increases (a & b). HAE incidence for the forward (c & d) and back (e & f) positional groups Shaded regions indicate 95% CI and n represents the number of players available for calculation based on compliance requirements (see Methods).


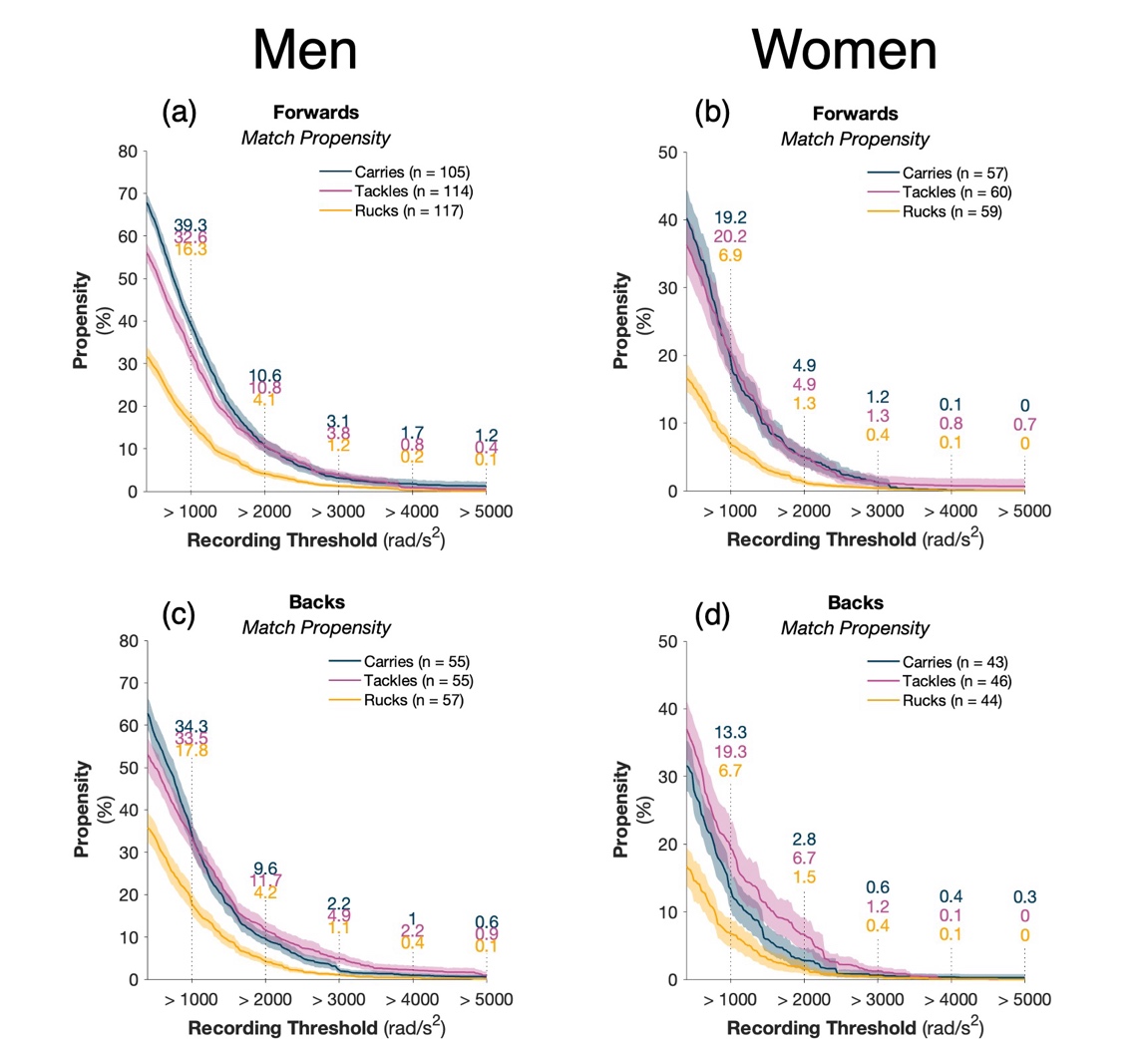


Supplementary Figure 2. The propensity of tackles, carries, and rucks for men and women to result in at least one HAE exceeding a given magnitude as PAA recording threshold increases for forwards (a & b) and backs (c & d). Shaded regions indicate 95% CI, and n represents the number of players available for calculation based on compliance requirements (see Methods).


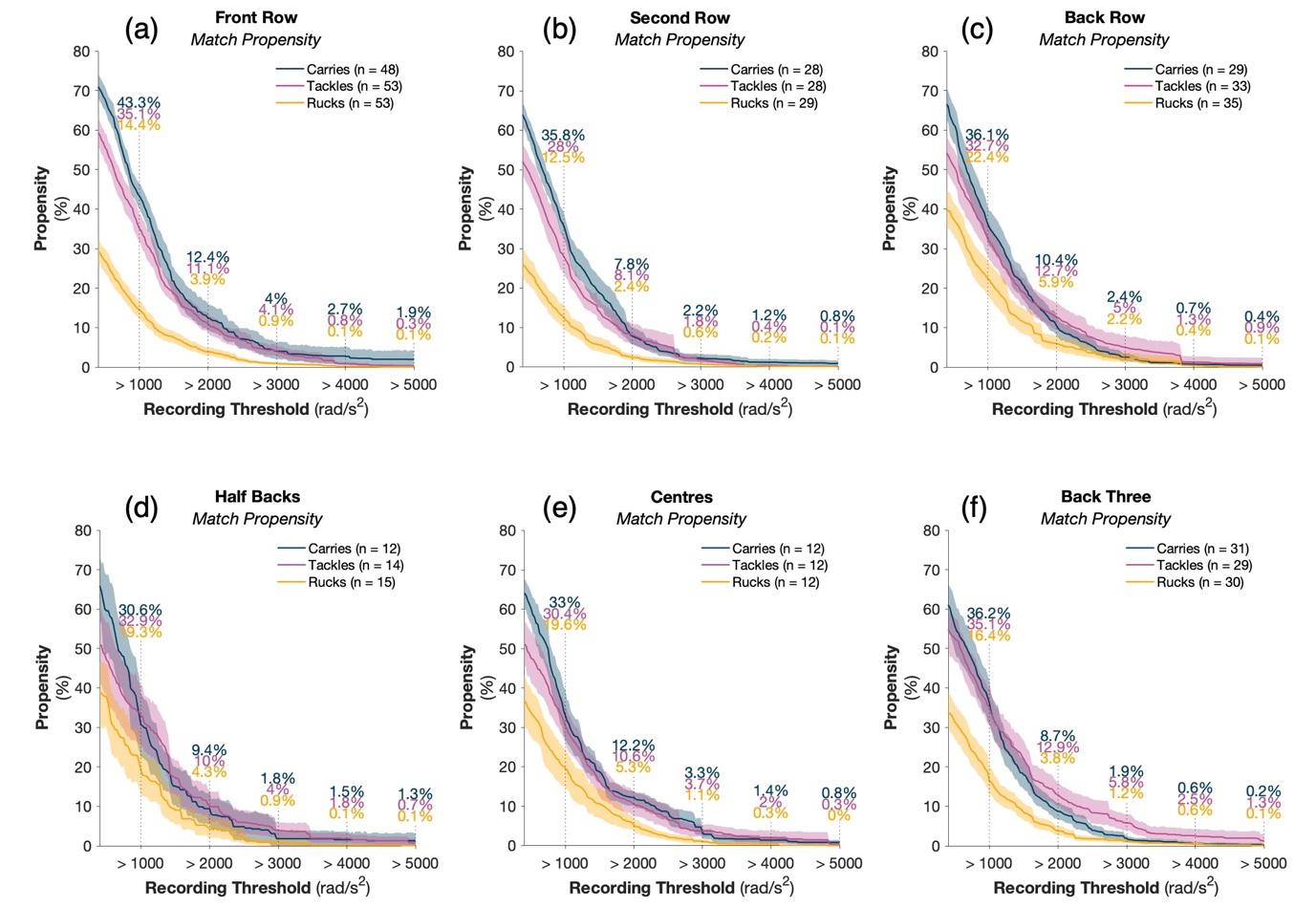


Supplementary Figure 3. The propensity of tackles, carries, and rucks for the men’s game to result in at least one HAE exceeding a given magnitude as PAA recording threshold increases for the Front Row (a), Second Row (b), Back Row (c), Half Backs (d), Centres (e), and Back Three (f) positional groups. Shaded regions indicate 95% CI and n represents the number of players available for calculation based on compliance requirements (see Methods).


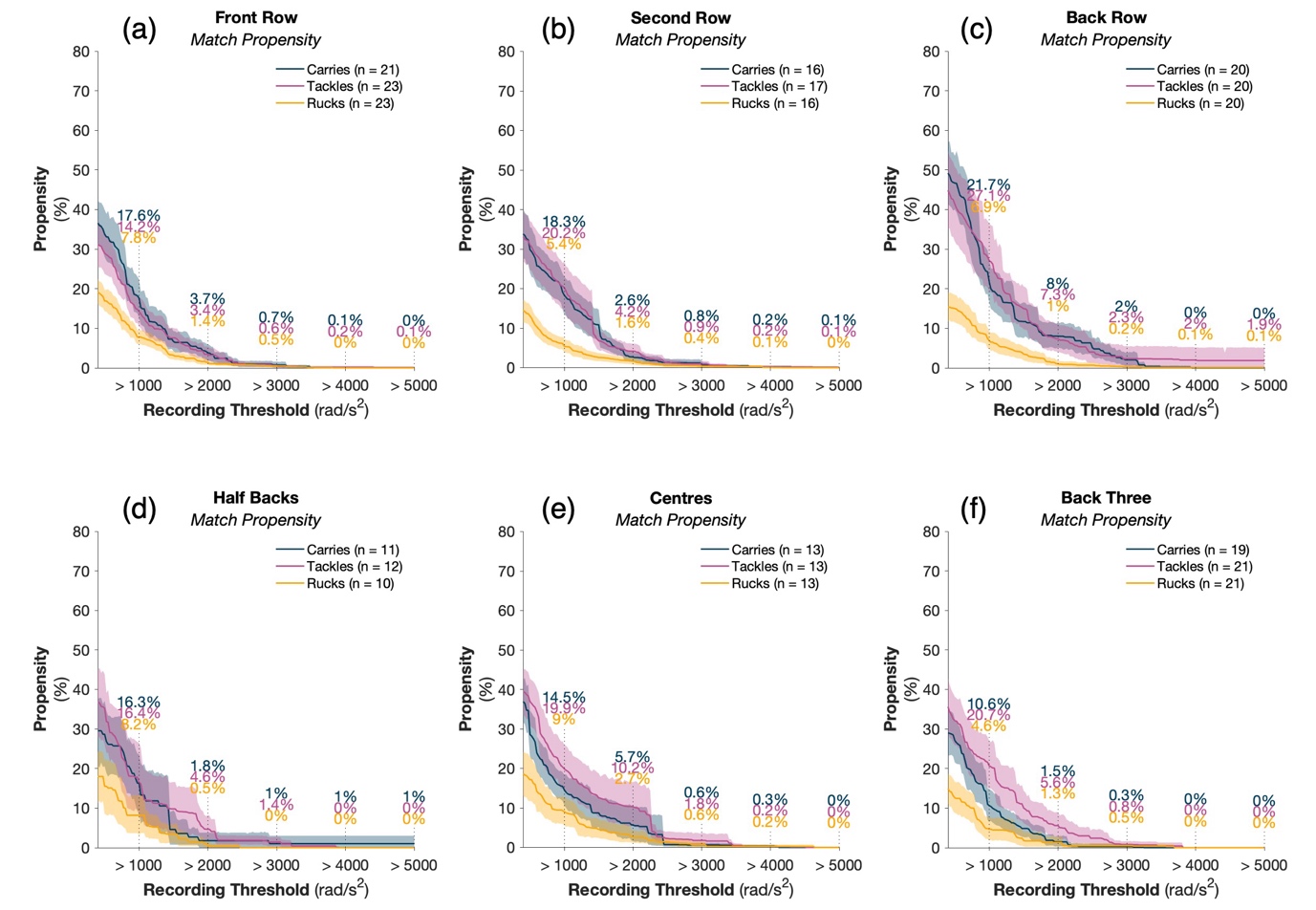


Supplementary Figure 4. The propensity of tackles, carries, and rucks for the women’s game to result in at least one HAE exceeding a given magnitude as PAA recording threshold increases for the Front Row (a), Second Row (b), Back Row (c), Half Backs (d), Centres (e), and Back Three (f) positional groups. Shaded regions indicate 95% CI and n represents the number of players available for calculation based on compliance requirements (see Methods).
